# Supplementary material for: Conspicuous carotenoid-based pelvic spine ornament in three-spined stickleback populations—occurrence and inheritance
Source: PeerJ. 2015 Apr 2;3:e872. doi: 10.7717/peerj.872 (PMC4389276; doi:10.7717/peerj.872)
Supplement: Appendix S1 — Recipe for feed given to stickleback offspring in the rearing experiment. [file peerj-03-872-s001.pdf]

1,110 g pellet-mix

207 g wheat fluor (boiled at 130° C in 20 min)

160 g oil from *Calanus sp.* (1.15 mg astaxantin/ml oil)

130 mg β,β-carotene

300 ml water

Dried over night at 30° C, and crushed into appropriate pieces.

Stored under nitrogen gas until use.
