# Supplementary figures and images for: Conspicuous carotenoid-based pelvic spine ornament in three-spined stickleback populations—occurrence and inheritance
Source: PeerJ. 2015 Apr 2;3:e872. doi: 10.7717/peerj.872 (PMC4389276; doi:10.7717/peerj.872)

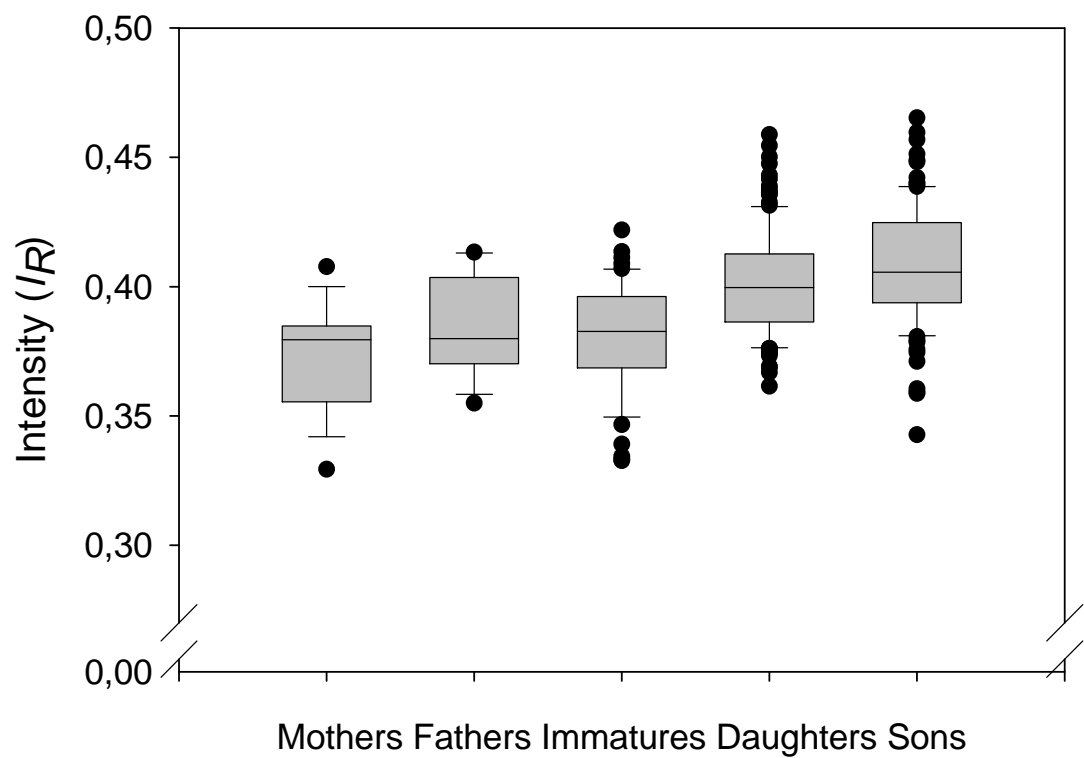

Supplement: Appendix S2 — Box–Whiskers plot of the intensity (IR) of the carotenoid-based ornament at the pelvic spines of the mothers (N = 15) and fathers (N = 15) in the artificial fertilizations, and their immature offspring (sex not identifiable, N = 55), daughters (N = 134) and sons (N = 103). Offspring IR was measured at an age of one year. Data are not adjusted for length. [file peerj-03-872-s002.pdf]

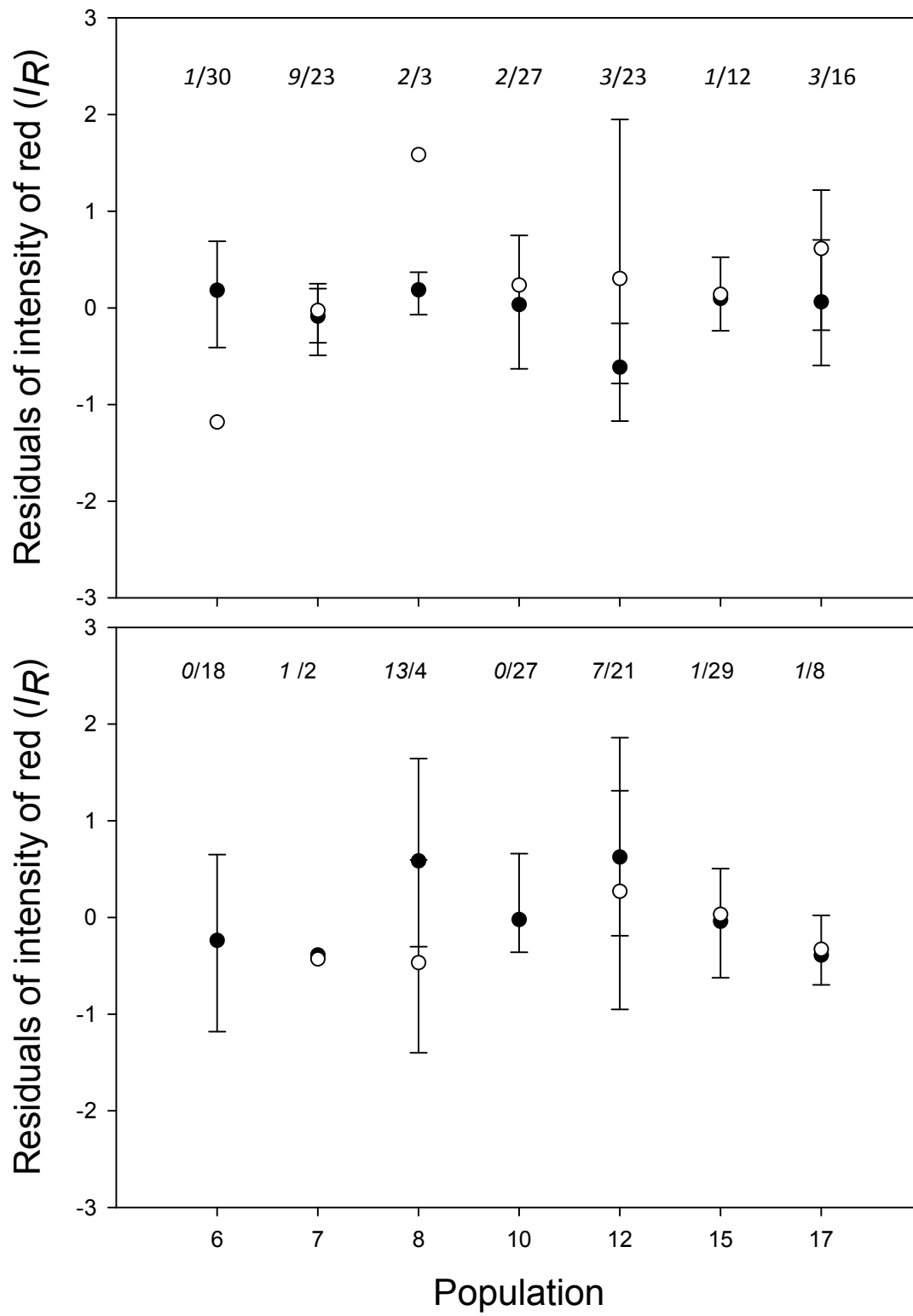

Supplement: Appendix S3 — Mean residuals of intensity of red (IR) of the pelvic spines of female (upper figure) and male (lower figure) sticklebacks, after adjusting for length of the fish. Fish infected by the tapeworm Schistocephalus solidus are shown as open circles, whereas uninfected ones are represented by filled circles. Lower and higher intervals shows 25 and 75%, respectively. Only populations with infected individuals are shown. Population numbers refer to Table 1. The numbers in the figure show number of sticklebacks examined: the first number (in italics) is number of sticklebacks infected, whereas the last number is number of fish not infected in each population. [file peerj-03-872-s003.pdf]
