# Supplementary material for: Conspicuous carotenoid-based pelvic spine ornament in three-spined stickleback populations—occurrence and inheritance
Source: PeerJ. 2015 Apr 2;3:e872. doi: 10.7717/peerj.872 (PMC4389276; doi:10.7717/peerj.872)
Supplement: Appendix S4 — All offspring (immature/unknown, males and females) from the rearing experiment. Test statistics from a GLM type III (adjusted) sums of squares (SS) with intensity of red colour of the offsprings’ pelvic spines (IR) as the response variable, and the predictors are “Offspring’s sex” (fixed factor), and offsprings’ “length”, IR of mothers (“Mothers’ IR”) and fathers (“Fathers’ IR”) as covariates. Adjusted R2 = 0.313. [file peerj-03-872-s004.pdf]

Source	SS	d.f	F	P-value
Analysis of variance				
Offsprings' sex	0.008	2	10.541	< 0.001
Length	0.017	1	46.300	<0.001
Mothers' I_R	0.002	1	6.676	0.010
Father's I_R	<0.001	1	0.120	0.730
Error	0.107	286		
Total	46.899	292		
