# Supplementary material for: Conspicuous carotenoid-based pelvic spine ornament in three-spined stickleback populations—occurrence and inheritance
Source: PeerJ. 2015 Apr 2;3:e872. doi: 10.7717/peerj.872 (PMC4389276; doi:10.7717/peerj.872)
Supplement: Appendix S5 — Sons from the rearing experiment: Test statistics from a GLM type III (adjusted) sums of squares (SS) with intensity of red colour of the offsprings’ pelvic spines (IR) as the response variable, and effects of “length” and IR of mothers pelvic spines (“Mothers’ IR”) as covariates. Adjusted R2 = 0.223. [file peerj-03-872-s005.pdf]

Source	SS	d.f	F	P-value
Analysis of variance				
Length	0.011	1	26.914	<0.001
Mothers' I_R	0.001	1	3.245	0.075
Fathers' I_R	3.05×10^{-5}	1	0.075	0.785
Error	0.040	99		
Total	17.231	103		
**Term**	**Coeff.**	**SE Coef.**	t	P-value
Coefficients				
Constant	0.252	0.0638	3.950	0.001
Length	0.002	<0.001	5.216	<0.001
Mothers' I_R	0.208	0.116	1.790	0.075
Fathers' I_R	-0.036	0.132	-0,273	0.785
